# Linking serum vitamin D levels with gut microbiota after 1-year lifestyle intervention with Mediterranean diet in patients with obesity and metabolic syndrome: a nested cross-sectional and prospective study

**DOI:** 10.1080/19490976.2023.2249150

**Published:** 2023-08-30

**Authors:** Hatim Boughanem, Patricia Ruiz-Limón, Jesús Pilo, José Manuel Lisbona-Montañez, Francisco J. Tinahones, Isabel Moreno Indias, Manuel Macías-González

**Affiliations:** a Department of Endocrinology and Nutrition, Virgen de la Victoria University Hospital, Malaga, Spain; b CIBER in Physiopathology of Obesity and Nutrition (CIBEROBN), Instituto de Salud Carlos III, Madrid, Spain; c Unidad de Gestión Clinica Medicina Interna, Lipids and Atherosclerosis Unit, Maimonides Institute for Biomedical Research in Córdoba, Reina Sofia University Hospital, Córdoba, Spain; d Department of Rheumatology, Regional University Hospital of Málaga, Malaga, Spain; e Department of Medicine and Dermatology, Faculty of Medicine, University of Malaga, Málaga, Spain

**Keywords:** 25-hydroxyvitamin D, obesity, metabolic syndrome, gut microbiota, vitamin D, MedDiet, mediterranean diet

## Abstract

Vitamin D, microbiota, and the Mediterranean diet (MedDiet) have been the focus of recent research due to their potential role in maintaining overall health. We hypothesize that MedDiet may alter the gut microbiota profile through changes in vitamin D levels. We aimed to investigate changes in gut microbiota and serum vitamin D levels after a MedDiet within a lifestyle intervention. The study included 91 patients with obesity and metabolic syndrome, who were categorized based on their serum vitamin D levels as having either optimal or low 25-hydroxyvitamin D [25(OH)D levels]. The profile of the gut microbiota was analyzed by the 16S rRNA sequencing, inferring its functionality through PICRUsT. Participants underwent a hypocaloric MedDiet and change in their lifestyle for 1 year, and the profile and functionality of their gut microbiota were evaluated by analyzing inter-individual differences in time. At baseline, gut microbiota profiles qualitatively differed between participants with Optimal or Low 25(OH)D levels [Unweighted (*p* = 0.016)]. Moreover, participants with Optimal 25(OH)D levels showed a higher gut microbiota diversity than those with Low 25(OH)D levels (*p* < 0.05). The differential analysis of abundance between the Low and Optimal 25(OH)D groups revealed differences in the levels of *Bacteroides*, *Prevotella*, and two *Clostridiales* features. After 1-year dietary intervention, both groups increased their 25(OH)D levels. Furthermore, both groups did not show significant differences in gut microbiota diversity, although the Low 25(OH)D group showed greater improvement in gut microbiota diversity by comparing at baseline and after dietary intervention (*p* < 0.05). Changes in specific bacterial taxa were observed within each group but did not differ significantly between the groups. Metabolic pathway analysis indicated differences in microbial functions between the groups (*p* < 0.05). These findings suggest that 25(OH)D status is associated with gut microbiota composition, diversity, and functionality, and lifestyle intervention can modulate both gut microbiota and 25(OH)D levels, potentially influencing metabolic pathways.

## Introduction

The Mediterranean Diet (MedDiet) is one of the healthiest dietary patterns and is associated with improved health and longevity.^1^ Recent studies have demonstrated that higher adherence to the MedDiet is associated with a lower risk of all‐cause mortality and a reduced incidence of a few chronic diseases including cardiovascular disease, type 2 diabetes, or cancer,^2^ and other metabolic disorders,^3^ such as obesity and components of the metabolic syndrome.^4^ Although many aspects need to be still deciphered, these positive effects have been mainly attributed to the beneficial and protective effects of polyphenols, flavonoids, and terpenoids present in the MedDiet. All these compounds have anti-inflammatory, antioxidative, antithrombotic, as well as lipid‐lowering properties.^5^ Furthermore, recent evidence has also demonstrated that MedDiet could influence the gut microbiota and microbial metabolites.^6^ According to a study conducted by Delgado-Lista *et al*. (2016), adherence to a MedDiet rich in olive oil is associated with increased levels of relative abundance of *Bacteroidetes, Clostridium cluster XIVa, Faecalibacterium prausnitzii, Lactobacilli, Roseburia*, and *Bifidobacteria*, as well as decreased levels of *Firmicutes*.^7^ This is potentially linked to a higher ability of fermentation to make short-chain fatty acids, which are advantageous metabolites for immunological and metabolic health.

Vitamin D is a fat-soluble vitamin that can act as a pleiotropic hormone and is an essential nutrient for health and the mitigation of many diseases, including obesity and cancer.^8^ Despite vitamin D’s primary function of maintaining bone and skeletal health, recent research has focused on its immune regulation because immune cells such as lymphocytes T and B and monocytes can express vitamin D receptor (VDR), and anti-inflammatory properties.^9^ Despite the importance of vitamin D in maintaining good health, surprisingly, the food included in the MedDiet does not offer a particularly high amount of vitamin D but it has the potential to enhance the bioavailability of vitamin D and cause changes in the composition of the gut microbiota, potentially leading to increased absorption of vitamin D.^10^ A cross-sectional study of healthy people who were administered vitamin D found a negative association with the relative abundance of *Prevotella* and a positive association with the relative abundance of *Bacteroides*.^11^ In contrast, another study showed that healthy participants with a high vitamin D intake have an increased fecal abundance of *Prevotella* and a lower abundance of *Haemophilus* and *Veillonella*.^12^ These differences between studies guarantee more research to understand how the microbiota can be influenced by exposure to increased circulating vitamin D levels and particularly by vitamin D deficiency. Moreover, while some studies have suggested that a MedDiet may promote healthy gut microbiota, it is unclear how much vitamin D levels contribute to this effect. Additionally, there is a limited understanding of the mechanisms by which vitamin D and the gut microbiota may interact.

Therefore, there is a need for further research to better understand the connections between vitamin D, and gut microbiota within a Mediterranean diet context, and to clarify the mechanisms involved. Based on this, we hypothesize that patients varying levels of vitamin D also exhibit differences in their gut microbiota populations. As a second hypothesis, we propose that MedDiet may alter the gut microbiota profile through changes in vitamin D levels. Therefore, the aim of this study was to investigate the gut microbiota profile of individuals with overweight/obesity and metabolic syndrome who have optimal or low levels of vitamin D, as well as to evaluate how these individuals vary their vitamin D levels and gut microbiota after 1-year of a lifestyle intervention using a MetDiet. Such research could have important implications for the development of interventions to improve health outcomes, particularly in the context of metabolic diseases.

## Results

### Gut microbiota comparison between low and optimal 25-hydroxyvitamin D groups

Anthropometric and biochemical parameters of Optimal 25(OH)D and Low 25(OH)D groups at baseline are presented in Table 1. We found no significant differences between the two groups, except for HDL levels. The Optimal 25(OH)D group had significantly higher levels of HDL than those with the Low 25(OH)D group (*p* = 0.011). Moreover, as expected, the Optimal 25(OH)D group consumed more dietary vitamin D than the Low 25(OH)D group (*p* = 0.050), while no significant differences were found in dietary calcium consumption. About pharmacological vitamin D, more participants in the Low 25(OH)D group were supplemented (*p* = 0.002).

**Table 1.** Summary descriptive table by groups according to 25-hydroxyvitamin D levels at baseline and 1-year.

Variables		Optimal 25(OH)D group (*n* = 45)	Low 25(OH)D group (*n* = 46)	*p-value* across groups^&^
Age (years), mean±SD		64.6 ± 4.47	64.7 ± 5.36	.910
Sex (male/female)		18/27	15/31	.463
Weight (Kg), mean±SD	Baseline 1-year	85.26 ± 1.67 81.46 ± 10.19*	86,72 ± 13.54 84.92 ± 14.01*	.569 .182
Waist circumference (cm), mean±SD	Baseline 1-year	111.92 ± 8.34 107.77 ± 9.26*	112.45 ± 9.92 110.69 ± 10.19*	.785 .157
Hip circumference (cm), median (IQR)	Baseline 1-year	112.50 (107.00–117.50) 109.00 (105.00–116.25)*	111.00 (106.67–12.00) 110.50 (104.37–117.87)*	.784 .721
BMI (Kg/m^2^, median (IQR)	Baseline 1-year	32.33 (3.54–36.30)31.07 (29.25–34.46)*	32.25 (29.94–36.06) 32.35 (29.63–35.80)	.803 .398
Glucose (mg/dL), median (IQR)	Baseline 1-year	109.00 (99.00–119.50)106.00 (99.00–114.00)	103.00 (94.75–117.75) 101.00 (92.00–110.25)*	.309 .077
HbA1c (%)	Baseline 1-year	5.94 ± .60 5.70 ± 1.02*	5.86 ± .80 5.73 ± 0.55	.558 .871
Triglycerides (mg/dL), median (IQR)	Baseline 1-year	135.00 (102.50–182.00)134.00 (98.00–176.00)	153.00 (121.00–186.50) 144.50 (112.25–191.25)	.309 .389
Total cholesterol (mg/dL), mean±SD	Baseline 1-year	203.84 ± 39.90 206.82 ± 40.32	192.52 ± 4.50 183.72 ± 35.07	.183 .004
HDL (mg/dL), median (IQR)	Baseline 1-year	53.20 ± 14.72 55.40 ± 14.47	46.20 ± 1.90 49.35 ± 10.60*	.011 .025
LDL (mg/dL), mean±SD	Baseline 1-year	121.09 ± 35.02 123.07 ± 34.17	114.30 ± 37.97 04.43 ± 31.17	.381 .008
25-hydroxyvitamin D (ng/mL), mean±SD	Baseline -year	32.14 ± 6.30 34.97 ± 8.37*	16.54 ± 5.13 29.99 ± 8.19*	<.001 .005
Dietary vitamin D (µg/day), median (IQR)	Baseline 1-year	6.91 (6.31–9.94) 6.76 (4.42–10.09)	6.63 (4.25–8.10) 7.44 (4.42–11.31)	.050 .582
Dietary calcium (mg/day), median (IQR)	Baseline 1-year	1135.85 (927.21–1358.87) 939.61 (759.03–1271.38)*	1088.21 (828.23–1424.32) 947.03 (749.13–1124.34)*	.721 .904
Participants with pharmacological vitamin D, (%)	Baseline 1-year	6.66 (3/45) 8.88 (4/45)	32.60 (15/46) 23.91 (11/46)	.002 .053

BMI, body mass index; HbA1c, glycated hemoglobin; HDL, high-density lipoprotein; IQR, interquartile range; LDL, low-density lipoprotein; SD, standard deviation. *p ≤ 0.05 baseline vs. 1-year of intervention value, according to paired Student’s tests or Wilcoxon tests. & Student’s t-test or Mann – Whitney test, Pearson’s chi-square test was used to calculate differences across groups.

**Figure 1.**
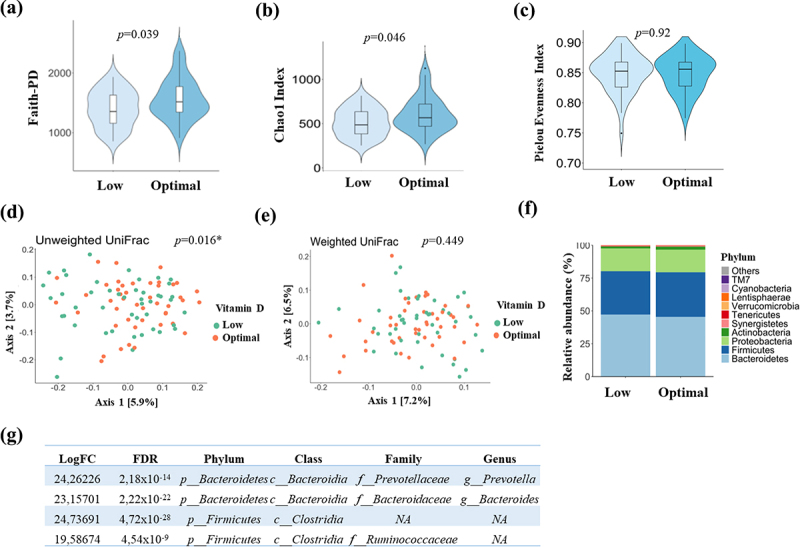
Gut microbiota analysis in Low and Optimal 25-hydroxyvitamin D groups at baseline. Alpha diversity indexes: a) Faith-PD; b) Chao1 index; and c) Pielou evenness index. Values are presented as mean ± SD. Principal coordinates analysis plot of d) Unweighted and e) Weighted UniFrac distance of fecal bacterial communities. f) bacterial abundances from the study groups at the phylum level. g) differential abundance analysis revealed statistically significant at family and genus levels between groups.

In terms of gut microbiota, we found that the Optimal 25(OH)D group had higher gut microbiota diversity than the Low 25(OH)D group, as indicated by significant differences in alpha diversity measured by Faith-PD (*p* = 0.039; Figure 1a) and Chao1 index (*p* = 0.046; Figure 1b). Regarding beta diversity, the two groups showed qualitative differences (Unweighted UniFrac Distance, *p* = 0.016; Figure 1 1d), although this difference was not significant quantitatively (Weighted UniFrac Distance, *p* = 0.449; Figure 1e). However, the plot networking showed that Low and Optimal 25(OH)D groups clustered into two distinct groups according to their microbiota profile (Supplementary Figure 1a), indicating differences between the groups. *Bacteroidetes, Firmicutes*, and *Proteobacteria* were the most prevalent phyla, with *Actinobacteria*, *Synergistetes, Tenericutes, Verrucomicrobia, Lentisphaerae, Cyanobacteria*, and *TM7* accounting for 1–10% of the total in both study groups (Figure 1f). The Low 25(OH)D group had a lower abundance of *Bacteroidetes* and a higher abundance of Firmicutes compared to the Optimal 25(OH)D group, which was also observed in the phylogenetic tree (Supplementary Figure 1b).

Moreover, the differential analysis of abundance between the Low and Optimal 25(OH)D groups revealed that the Low 25(OH)D group exhibited higher levels of *Bacteroides, Prevotella*, and two unknown features from the order *Clostridiales* than the Optimal 25(OH)D group (Figure 1g, Supplementary Figure 1c, Supplementary Table S1). To identify global shifts in microbial composition across the groups, we focused on the study of the core microbiota, which comprises taxa with a minimum relative abundance of 0.1% and is shared by 95% of the subjects. We identified four core microbiota, one for each time point, and visualized the common taxa between both groups. The core microbiota heatmap (Supplementary Figure 1d) displayed that families *Ruminococcaceae, Enterobacteriaceae, and Rikenellaceae*, as well as genera *Bilophila* and *Bacteroides*, were the five most prevalent taxa identified in the core microbiota analysis.

**Figure 2.**
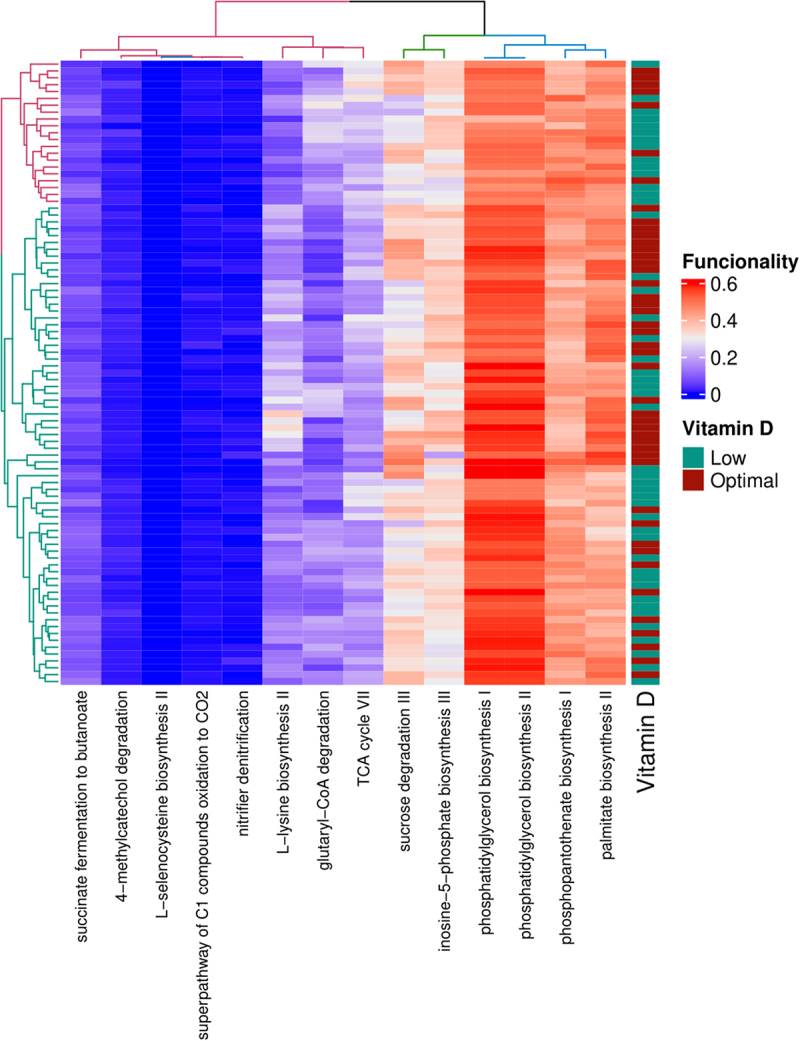
Heatmap representing mean values of significantly increasing or decreasing predicted metagenome pathways between Low and Optimal 25-hydroxyvitamin D groups. Predictive metabolic pathways were conducted by PICRUSt2. Kruskal Wallis test, *p* < .05.

On the other hand, further investigation using PICRUSt2 analysis revealed three different clusters of metabolic pathways that varied in their abundance and affected the analyzed groups differently. The less abundant cluster contained pathways related to degradation, utilization, and assimilation; fermentation to short-chain fatty acids (SCFAs); and L-selenocysteine biosynthesis. The second cluster contained L-lysine and purine nucleotide biosynthesis as well as carboxylate and carbohydrate degradation. The more abundant cluster included phospholipid, coenzyme A, and fatty acid biosynthesis (Figure 2). However, there was no clear distinction between the levels of vitamin D and their association with the different clusters.

### Changes in gut microbiota after 1-year lifestyle intervention and changes in 25-hydroxyvitamin D

To evaluate the dietary and lifestyle interventions, we summarized the anthropometric and biochemical parameters of the participants after 1-year of follow-up. The results are presented in Table 1. We observed that both groups, Optimal and Low 25(OH)D, decreased their weights (*p* < 0.001 and *p* = 0.008, respectively), waist (*p* < 0.001 and *p* = 0.029, respectively), and hip (*p* < 0.001 and *p* = 0.006, respectively) circumferences, as well as the dietary calcium (Optimal 25(OH)D group: *p* = 0.008; Low 25(OH)D group: *p* = 0.004). Moreover, both groups increased their 25(OH)D levels (Optimal 25(OH)D group: *p* = 0.021; Low 25(OH)D group: *p* < .001). However, BMI (*p* < .001) and HbA1c (*p* = .031) were only reduced in the Optimal 25(OH)D group, while the Low 25(OH)D group significantly decreased its glucose levels and increased its HDL levels (*p* = .004). In terms of pharmacological vitamin D, no differences were observed between the two groups after 1-year.

**Figure 3.**
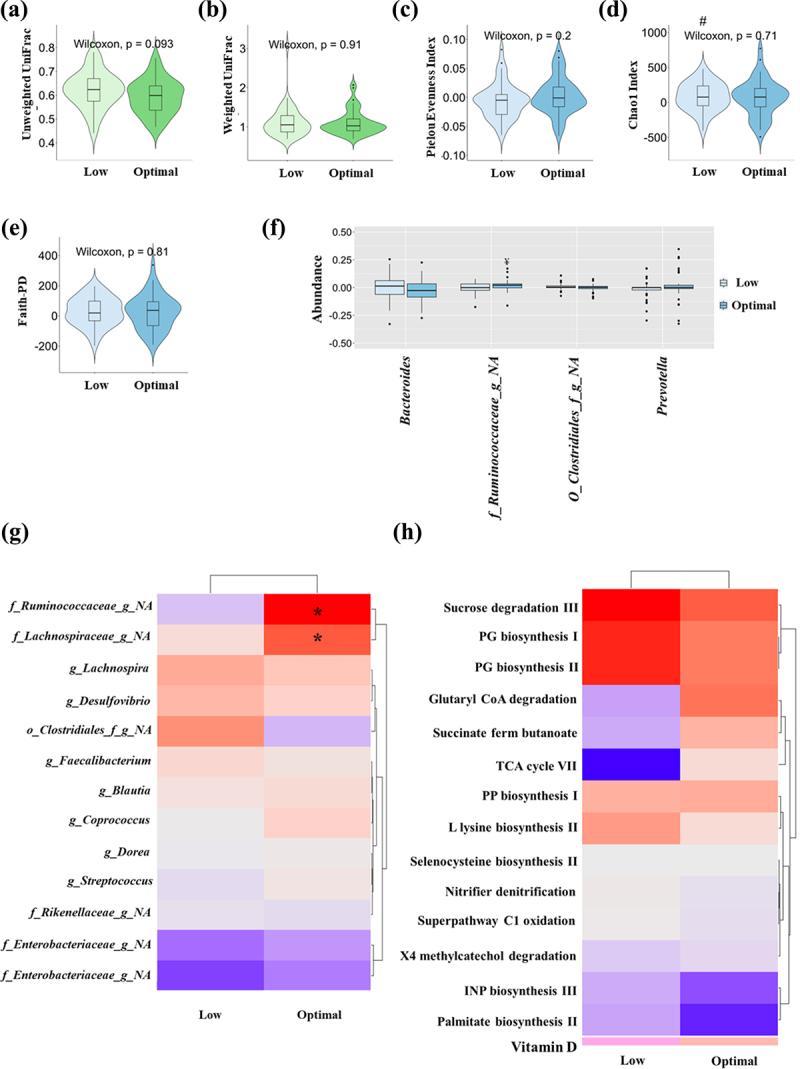
Gut microbiota analysis in Low and Optimal 25-hydroxyvitamin D groups after 1-year of the Mediterranean diet intervention. Beta diversity changes after 1-year of intervention with Mediterranean diet between the two groups: a) Unweighted and b) Weighted UniFrac. Alpha diversity changes after 1-year of intervention with Mediterranean diet between the two groups: c) Pielou evenness index, d) Chao1 index, e) Faith-PD. # indicates a significant difference between baseline and after 1-year intervention in the Low 25(OH)D group (*p* < .05, Wilcoxon test). f) changes in the genus abundance after 1-year with Mediterranean diet intervention between the two groups. ¥ indicates a significant difference between baseline and after 1-year intervention in the Optimal 25(OH)D group (*p* < .05, Wilcoxon test). g) Heatmap representing the gut microbiota abundance changes observed after 1-year with Mediterranean diet intervention. * indicates significant difference between Low and Optimal 25-hydroxyvitamin D groups (*p* < .05, Wilcoxon test). h) Heatmap representing the change in predicted metagenome pathways after 1-year of dietary intervention with Mediterranean diet between participants of the Low and Optimal 25-hydroxyvitamin D groups. Predictive metabolic pathways were conducted by PICRUSt2. Kruskal Wallis test, *p* < .05.

Focusing on changes in the gut microbiota, after 1-year of the MedDiet lifestyle intervention both groups, Low and Optimal 25(OH)D, increased their beta diversity, although the Low 25(OH)D group in the greatest manner in its qualitative version reached a statistical tendency (Unweighted UniFrac distance, *p* = .093; Figure 3a). However, in its quantitative version, by using the Weighted UniFrac distance, no differences were observed (*p* = .910; Figure 3b). In general, alpha diversity specifically increased in the Low 25(OH)D group with the intervention: evenness-Pielou (*p* = .081; Figure 3c), Chao1 index (*p* = .012; Figure 3 3d), and Faith-PD (*p* = .058; Figure 3e), while no significant differences were observed within the Optimal 25(OH)D group with the intervention. However, when changes between groups were assessed, no statistical changes were observed (*p* > .05).

The dominant bacterial phyla, *Bacteroidetes, Firmicutes, and Proteobacteria*, changed similarly, without significant differences between groups. However, the Optimal 25(OH)D group increases its abundance of *Firmicutes* (*p* = .002) and decreases its abundance of *Proteobacteria* (*p* = .010), while the Low 25(OH)D group only showed a statistical trend increasing its abundance of *Firmicutes* (*p* = 0.089; Supplementary Table S2).

About the bacteria that differed at baseline between the study groups, *Bacteroides*, *Prevotella*, *unknown features from the order Clostridiales*, and *unknown genus of Ruminococcaceae*, the intervention similarly modulated them without statistical differences between groups. Although *Bacteroides* and *unknown genus of Ruminococcaceae* showed a statistical tendency between changes in both groups (*p* = .1 and *p* = .053, respectively), the Optimal 25(OH)D group suffered a decrease in *Bacteroides* (*p* = .07; Figure 3f) and an increase in *unknown genus of Ruminococcaceae* (*p* = .001; Figure 3f).

Deeping into the results, an importance feature analysis was implemented to know the most important genera that were predictive of the intervention. Even though the accuracy model was not statistically significant (*p* > .05), those genera with at least un 0.2% of importance, included 13 genera and accounted for 0.52% of the total feature importance. These genera included: different genera of the family *Lachnospiraceae*, including *Lachnospira*, *Blautia, Coprococcus*, *Dorea*, and *unknown genus*; two different genera from the family *Ruminococcaceae*: *Faecalibacterium* and an *unknown genus*; *Desulfovibrio*, *Streptococcus*, two *unknown genera of the family Enterobacteriaceae*, an *unknown genus from the family Rickenellaceae*, as well as an *unknown genus of the order Clostridiales*. A paired-sample analysis was done for each one of these features with the greatest importance. From those, *the unknown genus of the order Clostridiales* and the *unknown genus of the order Lachnospiraceae* increased their levels in the Low 25(OH)D group (*p* = .032 and *p* = .027, respectively) and the *unknown genus from the family Rickenellaceae* decreased its levels in the Optimal 25(OH)D group (*p* = .038), although changes did not result in differences between groups. But interestingly, the genus *Coprococcus* and the *unknown genus of the family Ruminococcaceae* increased their levels in the Optimal 25(OH)D group (*p* = .011 and *p* = .001, respectively), and changes were statistically different between groups (*p* = .038 and *p* = .054, respectively; Figure 3g).

The metabolic pathways examined in both groups, Low and Optimal 25(OH)D, were further analyzed to explore the changes between the groups using pairwise analysis. The findings of this analysis are summarized in Figure 3h. According to the Kruskal Wallis test, there was a significant increase in the pathway succinate fermentation to butanoate in the Optimal 25(OH)D group after 1-year of intervention, compared to the Low 25(OH)D group (*p* = .018). Additionally, glutaryl-CoA degradation and TCA cycle VII tended to increase in the Optimal 25(OH)D group after 1-year of intervention, compared to the Low 25(OH)D group (*p* = .07 and *p* = .09, respectively).

## Discussion

Vitamin D is a critical factor that connects various processes within the human body. In the same way, the gut microbiota is also an important factor connecting different metabolic pathways. Interestingly, research has found a close association between vitamin D and changes in the intestinal microbiota.^13^ In our study, we found that optimal levels of vitamin D were associated with increased gut microbiota diversity, as well as specific gut microbiota profiles. Moreover, a MedDiet lifestyle intervention was able to increase serum vitamin D levels and gut microbiota diversity, similar to the Optimal 25(OH)D group. These findings suggest that vitamin D may have a positive effect on gut microbiota. However, the relationship is complex, and further research is needed to determine the extent of this effect.

In the current study, we focused on individuals with obesity and metabolic syndrome who had clinically low or optimal levels of 25(OH)D and studied their gut microbiota profiles. We found that gut microbiota populations (through beta diversity) differed significantly based on vitamin D adequacy. Furthermore, we found that the Optimal 25(OH)D group had a higher microbiota diversity, consistent with a previous study conducted on children by Singh *et al*.^14,15^ Higher levels of diversity are traditionally associated with better host health status, reflecting the capacity for adaptation to changing environments such as diet or health status.^16^ A posterior differential analysis revealed several features that differed between the groups: *Bacteroides* and *Prevotella*, as well as two members of the *Firmicutes* phylum and order *Clostridiales*, were all increased in the Low 25(OH)D group compared to the Optimal 25(OH)D group. The low number of differential abundance features may be due to the fact that vitamin D appears to have a greater effect on the gut microbiota in the upper gastrointestinal tract, with regional differences in the response of the gut microbiota.^17^ Therefore, further investigation is warranted to better understand this relationship.

Nevertheless, the differences observed in the current study are consistent with the scientific literature. The highest levels of *Bacteroides* in the low 25(OH)D group are consistent with several previous studies that assessed vitamin D and microbiota, but opposite to others.^14,18–21^ These controversial results may be related to the fact that *Bacteroides* is a genus with diverse species, some commensal, and others pathogenic.^22^ Different *Bacteroides* species have been implicated in the metabolization of bile acids, as well as Gram-positive bacteria such as *Clostridiales*, due to their bile salt hydrolase (BSH) capacity, and may be related to cholesterol levels.^23^ Thus, different species with the ability to deconjugate bile acids through their BSH capacity can alter the cholesterol levels of the host.^24^ Our patients differed in their HDL-cholesterol levels, although further investigation is necessary to clarify this issue.

On the other hand, *Prevotella* has also been previously reported to be more abundant in patients with low levels of dietary vitamin D intake.^15^ However, in a cross-sectional study of healthy individuals, vitamin D intake was negatively associated with the abundance of *Prevotella* but strongly positively associated with *Bacteroides*.^11^ Previous studies have reached contradictory conclusions regarding the role of *Prevotella* species. De Vadder *et al*. demonstrated that elevated *P. copri* abundance is associated with improvements in glucose metabolism and insulin sensitivity due to the production of succinate,^25^ while a study by Pedersen *et al*. suggested that *P. copri* abundance is associated with insulin resistance, presumably due to increased branched-chain amino acids (BCAA) biosynthesis.^26^ These conflicting findings may be related to the dietary patterns of our participants, which are further discussed.

After the 1-year intervention of a hypocaloric MedDiet within a lifestyle intervention, both groups increased their levels of 25(OH)D significantly, although the Low 25(OH)D group in a greater manner. However, differences in vitamin D concentrations persisted between the groups. Thus, the intervention helped to reach improved levels of vitamin D. Moreover, the anthropometric and some biochemical variables of the participants were improved. On the other hand, gut microbiota diversity was improved in the Low 25(OH)D group, with the elimination of differences between groups, indicating that MedDiet under a lifestyle intervention could act as a possible intervention to improve microbiota diversity. According to the differential abundance analysis, bacteria from the majoritarian phyla *Firmicutes* and *Proteobacteria* were the most affected by the intervention. Thus, *Firmicutes* was increased particularly in the Optimal 25(OH)D group (Low 25(OH)D group showed a tendency) at the expense of *Proteobacteria*. MedDiet has been already related to an increase of *Firmicutes*, particularly fiber-degrading bacteria.^27^ Moreover, *Proteobacteria* features have been related to dysbiosis processes,^28^ and MedDiet has been shown as a factor that is able to restore gut health.^29^ The most important bacteria within the intervention have been previously related to the Mediterranean diet consumption. Thus, *Lachnospiraceae* and *Ruminococcaceae* families and some of their genera have been already related to Mediterranean diet adherence,^30^ as well as the increase of *Faecalibacterium* abundance with a Mediterranean diet.^31^ Moreover, all these features have been identified as SCFAs producers, and SCFAs production has been proposed as the main pathway of the beneficial effects of this dietary pattern, together with the amelioration of intestinal permeability.^32^ Interestingly, our PICRUSt predicted metagenome analysis revealed an enrichment of the succinate fermentation to butanoate pathway in the Optimal 25(OH)D group after 1-year of intervention. This pathway is involved in the production of butyrate, one of the main SCFAs.

Two were the main features that varied significantly between groups, *Coprococcu*s and an *unknown genus of the family Ruminococcaceae*. *Coprococcus* together with *Faecalibacterium* are the two major butyrate producers of the *Firmicutes* phylum.^33^ A randomized clinical trial in vitamin D-deficient overweight or obese adults showed that increased levels of vitamin D were associated with a greater abundance of bacteria from the genus *Coprococcus*.^34^ Another study found that men with higher levels of 1,25(OH)2D (the metabolite active of vitamin D) and higher activation ratios of vitamin D were more likely to possess butyrate-producing bacteria, which are associated with better gut microbial health.^35^ Many metabolites from the gut microbiota regulate the expression of the vitamin D receptor (VDR), suggesting a link between the microbiome and vitamin D and specific SCFAs could be one of these regulations.^36^ SCFAs appear to mediate the increase of vitamin D with a MedDiet intervention due to the number of SCFAs producers’ features found to be important and/or increased. SCFAs could help to improve and maintain vitamin D levels together with the increase of microbial diversity. The fact that the Low 25(OH)D group higher increases in the diversity indexes and the Optimal 25(OH)D group showed higher changes in these SCFA producers could suggest that the Mediterranean diet within a lifestyle intervention could exert different effects on gut microbiota depending on the vitamin D levels of the host. These findings, together with the fact that many vitamin D supplementation trials fail to trigger the desirable health benefits, may suggest that a dietary intervention with a MedDiet could be more efficient in improving vitamin D levels than vitamin D supplementation alone. The fact that vitamin D is a nutrient could be behind this rationale.

In our study, we conducted a comprehensive analysis of the effect of a large, long-term lifestyle intervention involving an energy-reduced MedDiet and optimal serum vitamin D levels on gut microbiota. We observed several changes in the abundance of key genera, which may be related to the homeostasis of the host and its relationship with diet and vitamin D. While these results are interesting, we acknowledge several limitations that prevent us from establishing causality, including the observational design of the study within a normal clinical practice, which precluded more stringent interventions such as the pharmacological vitamin D supplementation if the practitioner deemed it appropriate or recommendations about an increment in the physical activity. Thus, this intervention should be taken as a lifestyle intervention within a real-life setting, something that we believe that also represents a strength of the study as many vitamin D supplementation trials have previously failed to trigger the expected desirable effects. Furthermore, about the technical methods, the nature of the 16S rRNA sequencing method limits the depth of microbiota analysis.

## Conclusion

In our study, we found that decreased 25(OH)D levels are associated with reduced microbiota diversity and alterations in key gut microbiota features such as *Bacteroides* and *Prevotella*, indicating a close relationship between vitamin D and the gut microbiome. Furthermore, after a 1-year lifestyle-based intervention, we observed that a hypocaloric MedDiet is able to improve 25(OH)D levels, particularly in those patients with low basal levels of 25(OH)D. In these participants, we also observed an increase in microbiota diversity, as well as changes in specific gut microbiota members, providing support for our observational findings. These results suggest that vitamin D is linked to an increased microbiota diversity through a MedDiet lifestyle intervention. Moreover, a MedDiet lifestyle intervention may be a promising approach for improving microbiota diversity and overall health status.

## Material and methods

### Study participants

In this study, 91 participants with overweight or obesity (body mass index (BMI) ≥ 27 and ≤40 kg/m2) and metabolic syndrome (according to the updated harmonized definition of the International Diabetes Federation and the American Heart Association, and National Heart, Lung, and Blood Institute^37^ were selected from the “Virgen de la Victoria” University Hospital (Málaga, Spain) between 2015 and 2017. Participants were divided into two groups based on their median serum vitamin D levels (25(OH)D; 25-hydroxyvitamin D) as Optimal 25(OH)D ˃ 24.05 ng/mL (*n* = 45), and as Low 25(OH)D ≤24.05 ng/mL (*n* = 46). Following 1-year of lifestyle changes with a hypocaloric MedDiet, physical exercise, and the administration of pharmacological vitamin D, if necessary, according to the medical practitioner decision depending on the usual clinical practice and the background of the participant. If needed, supplementation was done with cholecalciferol or calcifediol. Anthropometric and biochemical determinations and gut microbiota were analyzed at baseline and after 1-year of intervention. The exclusion criteria were: a previous history of cardiovascular disease, any chronic medical condition (cancer, inflammatory bowel disease, cirrhosis, etc.), acute infectious processes, psychiatric disorders, alcohol, and drug abuse, use of specific medications (cytotoxic agents, immune-suppressors, etc.), any food allergy to Mediterranean diet foods, and the use of antibiotic therapy, probiotics, or prebiotics in the previous three months. The participants gave their written informed consent. The study protocol and procedures were approved according to the ethical standards of the Declaration of Helsinki.

### Anthropometric, biochemical determinations, and samples collection

At the baseline and 1-year follow-up visits, waist circumference, weight, and height were measured. The body mass index (BMI) was calculated as kg/m^2^. Peripheral venous blood samples were obtained, at both time points, after 12 hours of fasting. Serum glucose, total cholesterol, triglycerides, and high-density lipoprotein (HDL) cholesterol were quantified by standard enzymatic methods. The low-density lipoprotein (LDL) cholesterol was calculated by the Friedewald equation.^38^ Glycated hemoglobin was determined using a chromatographic method, and 25-hydroxyvitamin D [ELISA kit (Immundiagnostik, Bensheim, Germany)] as previously described.^39^ Fecal samples were collected at baseline and 1-year time points in a sterile hermetic flask and immediately stored at − 80°C until analysis.

### DNA extraction and 16S sequences analysis

DNA extraction from stools was done with the QIAamp DNA stool Mini kit (Qiagen, Hilden, Germany) according to the manufacturer’s protocol. DNA concentrations were determined by absorbance at 260 nm (A260), and purity by determining the A260/A280 ratio with a Nanodrop spectrophotometer (Nanodrop Technologies, Wilmington, DE, USA). The Ion 16S Metagenomics Kit (Thermo Fischer Scientific, Waltham, MA, USA) was used to amplify the ribosomal 16S rRNA gene region. The kit includes two primer sets (V2-4-8 and V3–6, 7–9) that cover the corresponding hypervariable regions of the 16S rRNA region in bacteria. Sequencing the libraries was built with the Ion Plus Fragment Library kit (Thermo Fischer Scientific) and barcoded with the Ion Xpress™ Barcode Adapters kit (Thermo Fischer Scientific). Emulsion PCR and sequencing of the amplicon libraries were performed on an Ion 530 chip (Ion 530^TM^ Chip Kit) using the Ion Torrent S5™system, and the Ion 510™/520™/530™ Kit-Chef (Thermo Fisher Scientific) according to the manufacturer’s instructions. After sequencing, Torrent Suite™ Server software (Thermo Fisher Scientific) version 4.0 was used to base calling and run demultiplexing. Quality sequences were further translated into amplicon sequence variants (ASVs) using DADA2 with adapted parameters for Ion Torrent data^40^ within the open-source Quantitative Insights into Microbial Ecology (QIIME2, version 2022.2). Alpha and beta diversities, core microbiota, OTU abundance, plot network as well and plot tree were assessed using the *Phyloseq* package.^41^ Alpha-diversity was assessed through different indexes (Faith´s PD, pielou-evenness, and Chao1), while β-diversity was measured using UniFrac distances in its unweighted and weighted versions and tested with PERMANOVA. VSEARCH and the reference base Greengenes version 13_8 clustered at 97% of identity were used for the taxonomic assignment. Differential abundance analyses were assessed with the DESeq2 package (alpha = 0.001). The phylogenetic Investigation of Communities by Reconstruction of Unobserved States plugin (PICRUSt2) was used to predict metagenome function within QIIME2.^42^ The MetaCyc^43^ pathways were normalized within QIIME2 and further analyzed using the open-source software STAMP (Statistical Analysis of Metagenomics Profiles) with Welch’s t test option.^44^ The analyses of the most relevant taxa after 1-year of intervention were performed using a volatility analysis into the QIIME2. This volatility analysis, within the q2-longitudinal plugin^45^ into the QIIME2, was used to search for important features, which were posteriorly analyzed to establish statistical significance within the same plugin.

### Statistical analyses

Normality was assessed using the Kolmogorov-Smirnov test. The results are reported as mean ± standard deviation (SD) for normally distributed data, and as median and interquartile range (IQR) for non-normally distributed data. Categorical variables were presented as numbers (percentages). To compare numerical variables between the two groups, Student’s t-test or Mann-Whitney U test was employed depending on the normality of the data. Pearson’s chi-square test was used for categorical variables. For the bivariate analysis, paired Student’s t-test was conducted for continuous data, while the Wilcoxon test was applied for non-normally distributed data. Pearson correlation coefficients between variables were performed. Analyses and graphic representation were pointed out and performed using R v.3.5.1 software (Integrated Development for R. RStudio, PBC, Boston, MA, USA), and SPSS (15.0 version for Windows: SPSS, Chicago, IL, USA). The significance *p* value was set at *p* < .05.^46^

## Supplementary Material

Supplemental Material

Supplemental Material

Supplemental Material

Supplemental Material

## Data Availability

Due to the nature of this research, participants of this study did not agree for their data to be shared publicly, so supporting data is not available.
