# Supplementary figures and images for: Linking serum vitamin D levels with gut microbiota after 1-year lifestyle intervention with Mediterranean diet in patients with obesity and metabolic syndrome: a nested cross-sectional and prospective study
Source: Gut Microbes. 2023 Aug 30;15(2):2249150. doi: 10.1080/19490976.2023.2249150 (PMC10469434; doi:10.1080/19490976.2023.2249150)

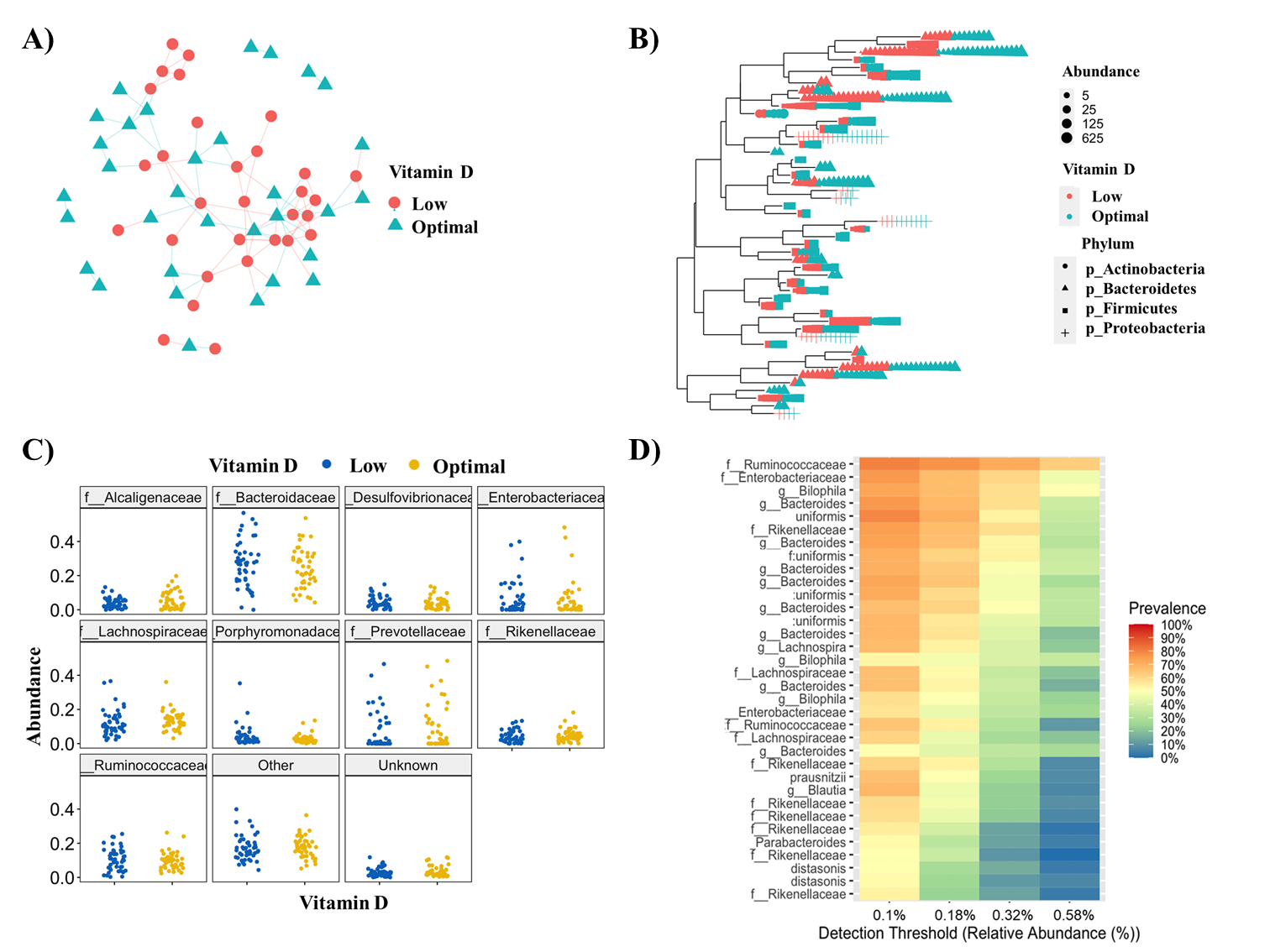

Supplement: Supplemental Material [file KGMI_A_2249150_SM3274.tif]
