# Supplementary material for: Linking serum vitamin D levels with gut microbiota after 1-year lifestyle intervention with Mediterranean diet in patients with obesity and metabolic syndrome: a nested cross-sectional and prospective study
Source: Gut Microbes. 2023 Aug 30;15(2):2249150. doi: 10.1080/19490976.2023.2249150 (PMC10469434; doi:10.1080/19490976.2023.2249150)
Supplement: Supplemental Material [file KGMI_A_2249150_SM3254.docx]

**Supplementary Figure 1.** A) Plot networking analysis of gut microbiota between participants of the Low and the Optimal 25-hydroxyvitamin D groups. B) Phylogenetic tree analysis of gut microbiota between participants of the Low and the Optimal 25-hydroxyvitamin D groups. C) Gut microbiota abundance analysis between the Low and Optimal 25-hydroxyvitamin D groups. D) Core microbiota analysis in participants of the Low and Optimal 25-hydroxyvitamin D groups. Heatmap depicting the core microbiome, their prevalence at different detection thresholds (relative abundance).
